# A three-dimensional flow model of screen channel liquid acquisition devices for propellant management in microgravity

**DOI:** 10.1038/s41526-022-00216-5

**Published:** 2022-07-28

**Authors:** Zheng Wang, Guang Yang, Ye Wang, Xin Jin, Rui Zhuan, Hao Zhang, Jingyi Wu

**Affiliations:** 1 Institute of Refrigeration and Cryogenics, Shanghai Jiao Tong University, Shanghai, 200240 China; 2 Joint Laboratory for Cryogenic Propulsion Technology of Aerospace Systems, Shanghai, 200240 China; 3 Aerospace System Engineering Shanghai, Shanghai, 201109 China

**Keywords:** Fluid dynamics, Chemical engineering, Mechanical engineering

## Abstract

Screen channel liquid acquisition devices (LADs) are among the most promising technologies for separating liquid and vapor phases in propellant storage tanks under microgravity conditions and thus ensuring vapor-free propellant supply to spacecraft engines. However, the prediction of the critical flow rate of a screen channel LAD relies on the full understanding of the three dimensional distribution of injection velocity. In this study, the flow characteristics at the entrance region of the LAD were investigated via particle image velocimetry (PIV) technique and numerical simulations under various working conditions. The experimental results illustrated that the velocity component normal to the porous woven mesh is non-uniform in both streamwise and spanwise directions of channel flow and that this phenomenon has a significant influence on the critical flow rate. Hence, a model that accounts for the three-dimensional flow field was proposed to predict the critical flow rate. The average error in the critical flow rate, which was determined by comparing the proposed model’s predictions and the experimental results, was less than 8.4%.

## Introduction

Due to the absence of acceleration under microgravity conditions^1^, it is challenging to guarantee vapor-free supply of propellant from a tank to an orbital spacecraft. The situation is even more serious for cryogenic propellants e.g., liquid oxygen (LOX) and liquid hydrogen (LH_2_), since their low boiling temperatures may accelerate evaporation and the fluids in the storage tank are usually in two-phase states^2–4^. In order to ensure effective propellant transportation, capillary-driven propellant management devices (PMDs) such as vanes, sponges, and screen channel liquid acquisition devices (LADs)^1,4–6^, which take full advantage of surface tension to separate vapor and liquid continuously without consuming excess energy, have been proposed. Among these, screen channel LADs are the most promising approach due to their applicability at relatively high flow rates and under adverse acceleration^2,7,8^. When a screen channel LAD operates, the liquid is driven by a pressure difference to flow through a porous woven mesh and down to an outlet. At the same time, the liquid within the microscopic mesh pores generates a capillary force that blocks vapor passage into the channel. Thus, the screen channel LAD can ensure that single-phase liquid is supplied to the engines.

The bubble point pressure (${{\Delta }}P_{{{{\mathrm{BP}}}}}$) and flow-through-screen pressure drop (${{\Delta }}P_{{{{\mathrm{FTS}}}}}$) are two critical parameters that govern LAD separation performance^9–11^. The bubble point pressure is the minimum pressure difference required for the vapor to break through the porous woven mesh and the flow rate at this pressure is defined as the critical flow rate. Experimental and theoretical analyses have verified a simplified bubble point model based on the Young–Laplace equation for room-temperature fluids and saturated cryogenic fluid states^4,12–15^. In general, the bubble point is determined from the effective pore diameter, surface tension and contact angle^12^. The relevant relationship is expressed as ${{\Delta }}P_{{{{\mathrm{BP}}}}}\sim 4\gamma \cos \theta _{{{\mathrm{c}}}}/D_{{{\mathrm{p}}}}$. In addition, Hartwig et al.^16^ found that the bubble point pressure was dominated by the liquid temperature, since higher surface tensions could be usually obtained at lower temperatures.

The flow-through-screen pressure drop (${{\Delta }}P_{{{{\mathrm{FTS}}}}}$) refers to the pressure loss that occurs when liquid flows across a wetted porous woven mesh. Modeling-based ${{\Delta }}P_{{{{\mathrm{FTS}}}}}$ prediction has been studied extensively for decades^17,18^, with related experiments conducted for both room-temperature and cryogenic fluids, including LH_2_, LN_2_, and H_2_O^19,20^. Armour and Cannon^18^ developed an empirical model that treated the porous woven mesh as a thin packed bed. McQuillen et al.^21,22^ conducted a series of numerical studies to explore LAD performances in various orientations and submersion depths based on the assumptions made by Armour and Cannon^18^. Hartwig et al.^23^ investigated LH_2_ and LOX pressure distributions inside the LAD channel experimentally. The results showed that the flow-through-screen (FTS) pressure drop is related to temperature and increases significantly at lower temperatures. Thereafter, Wang et al.^24^ developed an analytical model for the FTS pressure drop that considers the effects of pore structures on the flow.

A higher bubble point pressure requires a smaller pore diameter; conversely, a system with a lower FTS pressure drop prefers a larger pore size. There is an inherent trade-off between the FTS pressure drop and bubble point pressure when choosing the porous woven mesh. Therefore, it is necessary to achieve a compromise between these two parameters in order to optimize the LAD design. Previous studies focused mainly on porous woven mesh performance. Although some device-level experiments have been conducted using LADs^1^, the results focused on operational parameters, such as the flow rate and breakdown condition. Furthermore, due to the complexity of porous media flow, most of the traditional models assume a uniform injection velocity along the LAD channel, which results in an overprediction of the critical flow rate. Hartwig et al.^23^ proposed a one-dimensional (1D) steady-state model that assumed that the injection velocity was uniform throughout the porous woven mesh. The results were reported to overpredict performance by 18% compared with actual anti-gravity liquid acquisition tests. They also pointed out that a two-dimensional (2D) or three-dimensional (3D) model is required to estimate the injection velocity distribution along the LAD channel accurately. Among the limited studies that considered the non-uniformity of injection velocity, Hartwig and Darr^25^ and Darr et al.^26^ derived a mathematical solution from the 2D Navier–Stokes equations to predict the pressure drop distribution along the channel. Their 2D model was found to perform better than the previous 1D model. However, the velocity non-uniformity in the spanwise direction has not been taken into consideration. Moreover, there is still a lack of experimental data regarding the 3D injection velocity distribution to support the available theoretical models. Therefore, a detailed experimental investigation of flow dynamics through the porous woven mesh of a screen channel LAD is of great significance.

In order to solve the aforementioned problems, we investigated the characteristics of flow through the porous woven mesh of a screen channel LAD under various working conditions using particle image velocimetry (PIV) and numerical simulations. The detailed 3D velocity fields at the entrance region of the screen channel LAD are experimentally obtained. Based on the analysis of the velocity distribution, we propose a 3D flow model that can more accurately predict the critical flow rate of the screen channel LAD.

## Results

### The velocity field in the liquid acquisition system

**Fig. 1:**
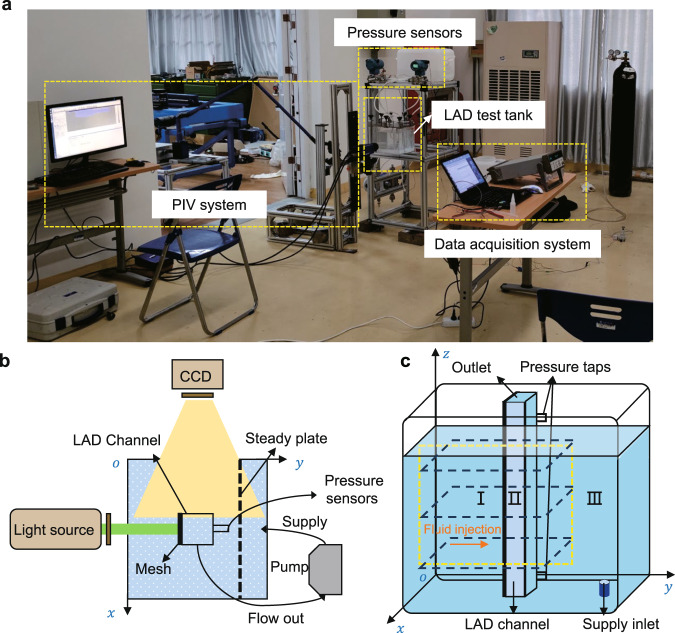
The anti-gravity liquid acquisition test system. **a** Photograph, **b** top-view schematic of the test tank when measuring the velocity at the *yz* plane, and **c** areas recorded using the PIV technique (dashed rectangles at the *yz* and *xy* planes). The recorded *yz* plane was divided into three zones. Zone I was the entrance zone, zone II was the channel zone, and zone III was the supply zone.

To investigate the fluid dynamics through the porous woven mesh of the screen channel LAD, an anti-gravity liquid acquisition test system was implemented. The system consisted of a test tank, a PIV facility, and a data acquisition system. Figure 1a, b presents a schematic of the experimental system. The recorded areas on *xy* and *yz* planes using the PIV technique are illustrated in Fig. 1c. Three types of Dutch Twill screens were used in the experiments (Supplementary Fig. 2), and their properties are shown in Table 1. Details on the theoretical basis, materials, experimental setup, and the data acquisition and reduction procedure are presented in the Methods section.

**Table 1:** Properties of porous woven mesh.

Mesh type	80 × 700 DT	130 × 1100 DT	165 × 1500 DT
Shute wire diameter (μm)	76	48	33
Wrap wire diameter (μm)	101	68	61
Porosity *ε*	0.370	0.369	0.418
Hydraulic pore diameter *D*_h_ (μm)	46.5	29.7	26.9
Effective pore diameter *D*_p_ (μm)	53.4 ± 5.8	35.2 ± 3.7	29.9 ± 3.1
Specific surface area S_v_ (m^−1^)	31842	49731	62148
*A*_p_ (m^−1^) of Eq. (13)^24^	6.4 × 10^6^	10.1 × 10^6^	6.9 × 10^6^
*B*_p_ of Eq. (13)^24^	13	13	7

**Fig. 2:**
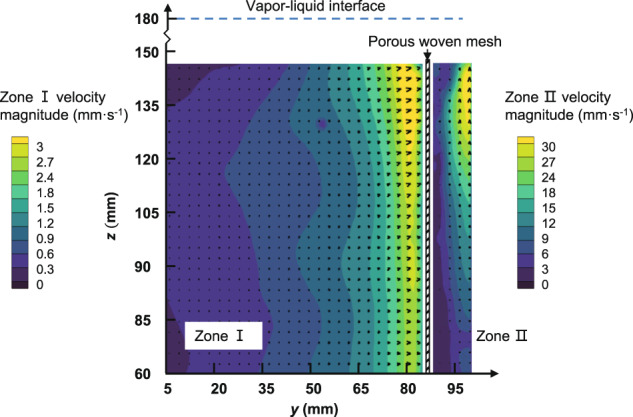
The distribution of the velocity vector at *yz* plane for the 80 × 700 DT mesh at *Q* = 43 L h^−1^. The velocity in zone II is generally one order of magnitude larger than that in zone I.

Figure 2 shows a typical velocity field at the *yz* plane for the 80 × 700 DT mesh at *Q* = 43 L h^−1^ by experiments. The distribution of the injection velocity illustrates that the fluid flows towards the *y* direction in zone I and then flows across the porous woven mesh. Afterwards, the fluid flows in the channel towards the outflow port at the top of the channel. It is obvious that the velocity in zone II (inside the channel) is one order of magnitude larger than that in zone I (the entrance region outside the channel) due to the large ratio between the inlet area of the submerged mesh and the cross-section of the channel. The velocity magnitude increases along the *y* and *z* directions in zone I and zone II and reaches its maximum near the outflow port inside the channel wall. The increase in the injection velocity in the *z*-direction results in a reduction of the critical flow rate, which is discussed further in this work.

### Distribution of injection velocity in the streamwise direction

**Fig. 3:**
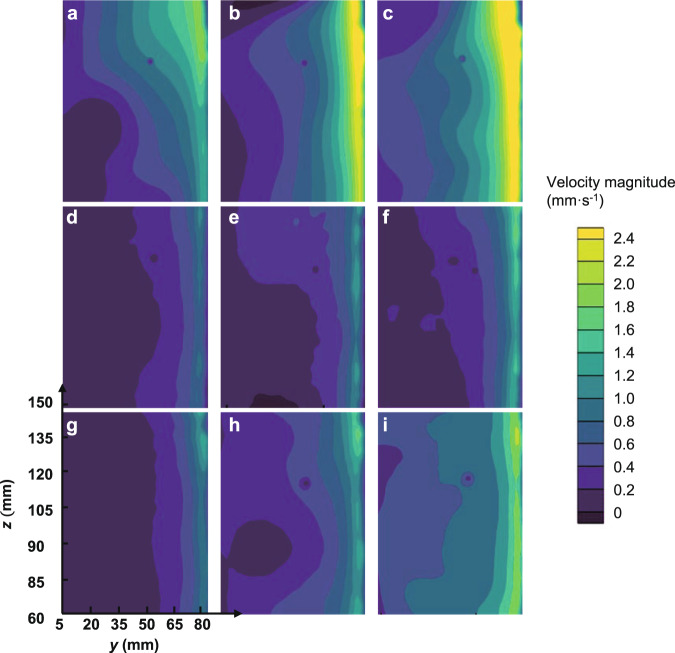
The velocity distribution in zone I at *yz* plane for various flow rates and porous woven mesh types. First row: 80 × 700 DT, second row: 130 × 1100 DT, third row: 165 × 1500 DT. First column: *Q* = 20 (±3) L h^−1^, second column: *Q* = 32 (±3) L h^−1^, third column: *Q* = 43 (±3) L h^−1^.

Velocity distributions for the various meshes and flow rates at the *yz* plane are shown in Fig. 3. All of the flow fields exhibit similar characteristics in zone I. In particular, the velocity at the entrance region increases along the *z* direction. For each case, higher values of velocity magnitude are mainly distributed in the vicinity of the mesh, and the velocity decreases rapidly with the increasing distance to the mesh. This also proves that the distance between the porous wall and the inner wall of the tank has little effect on the inlet velocity distribution if it is larger than 10 mm, as mentioned in the Methods section. For a given porous woven mesh, a higher outflow rate results in a larger velocity magnitude at the entrance region, and generates a larger velocity difference as well.

**Fig. 4:**
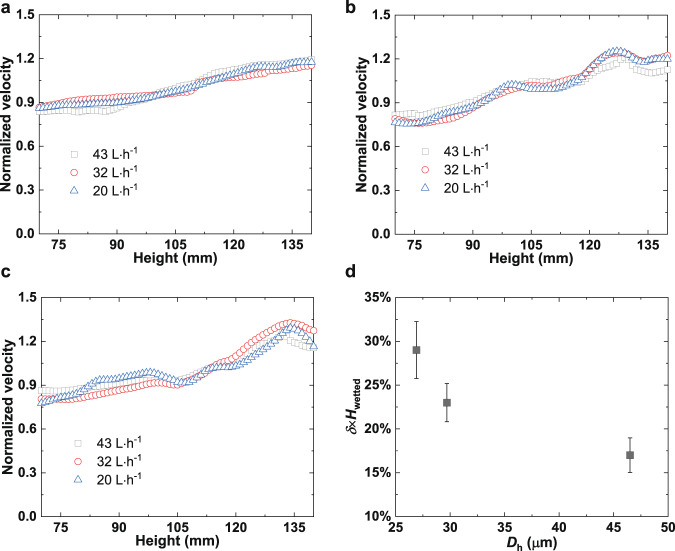
The distribution of normalized injection velocity for various porous woven meshes at different flow rates. **a** 80 × 700 DT, **b** 130 × 1100 DT, and **c** 165 × 1500 DT. **d** Injection velocity non-uniformity for various types of porous woven mesh (The error bar indicates the standard deviation of four independent experiments).

The injection velocity *v* at the centerline (*x* = 105 mm, *y* = 83 mm, *z* from 70 to 140 mm) before the porous woven mesh was extracted from the captured images to study the velocity variation at the entrance region, as shown in Fig. 4. In order to evaluate the velocity distributions in different cases the velocity was scaled, and the normalized velocity v^*^ was defined as follows:

1 $$v^ \ast = v/v_{{{{\mathrm{avg}}}} - {{{\mathrm{center}}}}},$$

where $v_{{{{\mathrm{avg}}}} - {{{\mathrm{center}}}}}$ is the line-averaged injection velocity at *y* = 83 mm and *x* = 105 mm, which can be calculated using $v_{{{{\mathrm{avg}}}} - {{{\mathrm{center}}}}} = \left( {{\int}_{x = W/2} {vdz} } \right)/H$.

The results indicate that the normalized injection velocity increases approximately linearly along the outflow direction in all cases, as shown in Fig. 4a–c. Therefore, a non-uniformity coefficient *δ* is utilized to describe the non-uniformity of the injection velocity at unit length in *z*-direction quantitatively,

2 $$\delta = \frac{{v^ \ast _{{{\mathrm{m}}}} - v^ \ast _{{{{\mathrm{avg}}}} - {{{\mathrm{center}}}}}}}{{H_{{{{\mathrm{wetted}}}}}}},$$

where $v_{{{\mathrm{m}}}}^ \ast$ is the normalized maximum injection velocity while $v_{{{{\mathrm{avg}}}} - {{{\mathrm{center}}}}}^ \ast$ is the normalized line-averaged injection velocity, and $H_{{{{\mathrm{wetted}}}}}$ is the length of the porous woven mesh that contacts the bulk liquid in the tank. The injection velocity non-uniformity for the entire fluid entrance region is calculated as $\delta \cdot H_{{{{\mathrm{wetted}}}}}$. Figure 4a–c shows that $\delta \cdot H_{{{{\mathrm{wetted}}}}}$ varies by less than 3%, when the flow rate is changed in the range of 20–43 L h^−1^ for each porous mesh. To evaluate the influence of mesh types on the injection velocity distribution, the injection velocity non-uniformity is calculated for 80 × 700 DT, 130 × 1100 DT, and 165 × 1500 DT meshes. As presented in Fig. 4d, the velocity non-uniformity decreases as the pore diameter increases. The injection velocity produced using an 80 × 700 DT (the largest pore size) is more uniform than that experienced with the smallest pore size (165 × 1500 DT). This also agrees with theoretical analysis (Supplementary Discussion). Other parameters affecting the velocity non-uniformity include the fluid properties, size, shape, and surface roughness of the LAD channel (Supplementary Discussion, Supplementary Equation 14).

### Distribution of injection velocity in the spanwise direction

**Fig. 5:**
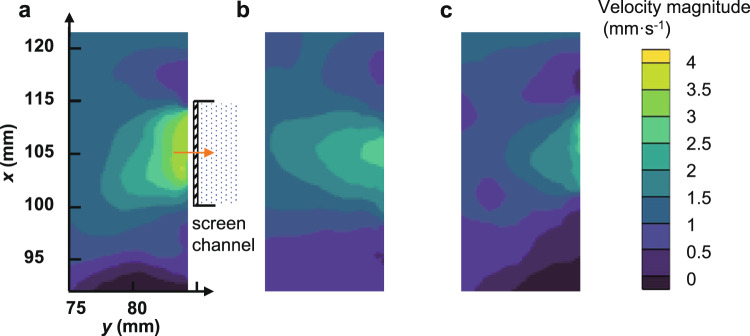
The distribution of injection velocity at different height levels. **a** *z* = 160 mm **b** *z* = 100 mm **c** *z=* 40 mm.

In order to explore the injection velocity distribution in the spanwise direction, the velocity field at *xy* planes of different height levels is also experimentally analyzed. Figure 5 shows the velocity distribution with a 90% fill level for 80 × 700 DT at *Q* = 43 L h^−1^. The velocity field at different height levels shows that the injection velocity close to the channel outlet is generally larger than that near the dead-end of the LAD channel, which is in accordance with the results shown in Fig. 2. Moreover, the experimental results indicate that the injection velocity at the middle of porous mesh is also larger than that near the side walls in *x* direction, which is a clear evidence that the injection velocity in *x* direction is also non-uniform. Similarly, a dimensionless scale factor *λ* could be utilized to quantitatively describe the non-uniformity of the injection velocity in *x*-direction, which is calculated as follow:

3 $$\lambda = \left( {{\int}_{x = W/2} {vdz/H} - {\int} {{\int}_{\begin{array}{*{20}{c}} {0 \le x \le W} \\ {0 \le z \le H} \end{array}} {vdxdz/WH} } } \right)/\left( {{\int} {{\int}_{\begin{array}{*{20}{c}} {0 \le x \le W} \\ {0 \le z \le H} \end{array}} {vdxdz/WH} } } \right).$$

The difference between the injection velocity at *x* = *W*/2 and the average velocity at the whole inlet can be obtained from the velocity field.

### Numerical simulations of the injection velocity field

In order to analyze the 3D flow behaviors in detail, numerical simulations of single-phase outflow in the LAD channel were performed. The computational domain was half (*H* = 200 mm, *L* = 15 mm, *W* = 7.5 mm) of the LAD channel with surrounding liquids, which is symmetrical at the vertical plane denoted by *x* = 105 mm. The governing equations were solved using a pressure-based SIMPLE algorithm and steady implicit formulation. Since the pore-scale Reynolds number ($Re = \rho u/\mu S_{{{\mathrm{v}}}}^2D_{{{\mathrm{p}}}}$) was smaller than 1 and the channel-scale Reynolds number ($Re = \rho uW/\mu$) was smaller than 1000 for the range of parameters considered in this work, the laminar flow model was used^27,28^. The second order scheme was used for pressure discretization. Furthermore, a second-order upwind scheme was used to discretize the momentum terms. The inlet and outlet conditions were set as the pressure-inlet and mass-flow-outlet, respectively. All of the solid walls are set as no-slip walls. The simulations were run using commercial CFD software, ANSYS Fluent.

The porous-jump model was used for the porous woven mesh^22^ since the mesh is thin (less than 1 mm) and the fluid flow is perpendicular to the mesh. In this model, the pressure gradient in the porous woven mesh is described using

4 $$dp/dy = D_y\mu v + \rho C_yv^2/2,$$

where $D_y$ is the viscous resistance coefficient and $C_y$ is the inertial resistance coefficient^21^. The pressure gradient inside the porous woven mesh is treated as constant, so the FTS pressure drop can be calculated as

5 $${{\Delta }}P_{{{{\mathrm{FTS}}}}} = \left( {D_y\mu v + \rho C_yv^2/2} \right){{\Delta }}m,$$

where ${{\Delta }}m$ is the thickness of the porous woven mesh^28^. According to Eq. (5), three parameters must be determined in order to use the porous-jump model: the face permeability $1/D_y$, the porous medium thickness ${{\Delta }}m$, and the porous-jump coefficient $C_y$. Equations (13) and (5) can be used to calculate these parameters for each porous mesh via the analytical model^24^. A total of 1.4 × 10^6^ hexahedral cells were used in all cases. Grid independence was confirmed by changing the number of cells from 1.4 × 10^6^ to 1.8 × 10^6^ and observing that the velocity deviation was smaller than 1%.

**Fig. 6:**
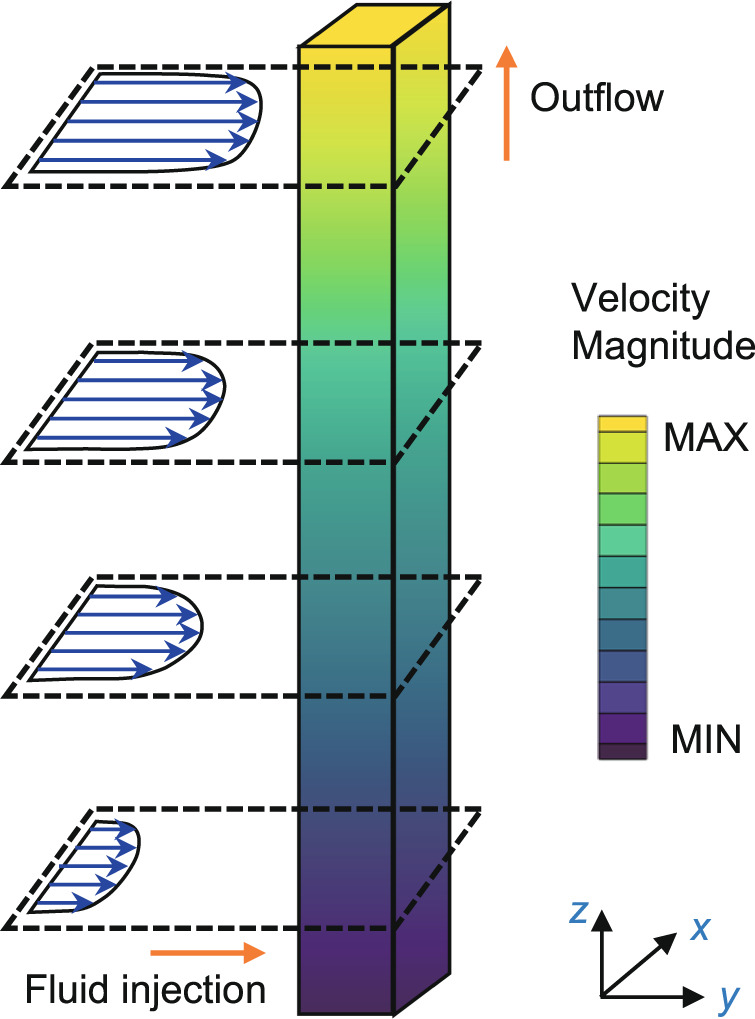
Typical velocity distributions at the entrance region and inside the LAD channel by numerical simulation. The velocity magnitude increases in the *z* direction along the channel and the injection velocity profile is approximately parabolic in the *x* direction.

**Fig. 7:**
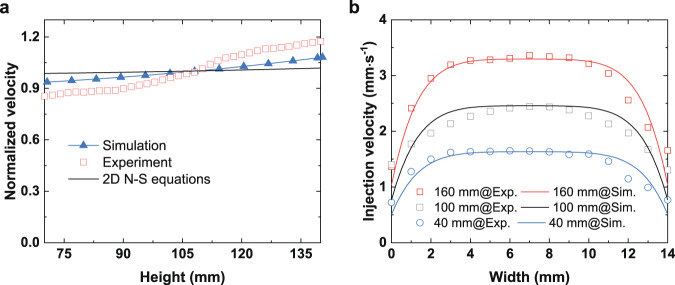
The distribution of injection velocity for simulation and experiments at *Q* = 43 L h^−1^ with 80 × 700 DT mesh. **a** Injection velocity at *yz* plane, **b** injection velocity at *xy* plane.

Typical velocity distributions at the entrance region and inside the LAD channel by the numerical simulations are shown in Fig. 6. That is, the velocity magnitude increases in the *z* direction along the channel and the injection velocity profile is approximately parabolic in the *x* direction. Simulated and experimentally determined injection velocity distributions along the *z* direction at the entrance region are compared in Fig. 7a for the flow rate of *Q* = 43 L h^−1^ and 80 × 700 DT mesh. The simulation results and experimental data exhibit a similar tendency as both increase along the *z* direction. The experimentally determined injection velocity non-uniformity in *z* direction is ~17.2%, while the simulation result is about 8.7%. The velocity distribution in the *z* direction from the present study is also found to be of the same trend but slightly more non-uniform as compared to that derived from the 2D Navier-Stokes equations^26^. (Supplementary Methods). The discrepancy between the experiment and simulation might be caused mainly by the assumption that the inner wall of the LAD channel is smooth in the simulations. Since the friction loss is smaller in the simulation, a smaller injection velocity non-uniformity would be observed. (Supplementary Discussion, Supplementary Equation 14)

The distributions of injection velocity in *x* direction at *z* = 40 mm, 100 mm, and 160 mm from the experiments and simulations are extracted and presented in Fig. 7b for the flow rate of *Q* = 43 L h^−1^ and 80 × 700 DT mesh. The average difference between experiments and simulations is about 3.4%, which further verified the present numerical model. Experimental results in Fig. 7b indicate that the non-uniformity factor of the injection velocity in *x*-direction, i.e., *λ* as defined in Eq. (3), changes slightly at various height levels, which are 19.2%, 18.5%, and 18.4% at *z* = 160 mm, 100 mm and 40 mm, respectively. The value of *λ* is 16.0% as calculated from the simulation results at *Q* = 43 L h^−1^ with 80 × 700 DT mesh, which is close to the experiments. The value of *λ* was also found to be less sensitive to the variation of the specification of the mesh.

### Effect of injection velocity field on the critical flow rate

As the injection velocity of the screen-channel LAD is non-uniform in both *x* and *z* directions, its effect on the critical flow rate is analyzed. When the critical maximum injection velocity is recorded as the average injection velocity, which is the so-called 1D model, the critical flow rate of a screen channel LAD is calculated as follows:

6 $$Q_{{{{\mathrm{cr}}}} - 1{{{\mathrm{D}}}}} = v_{{{{\mathrm{max}}}}} \cdot A_{{{\mathrm{c}}}}.$$

where $A_{{{\mathrm{c}}}}$ is the effective flow area of the porous woven mesh and $v_{{{{\mathrm{max}}}}}$ is the maximum injection velocity on $A_{{{\mathrm{c}}}}$ under the critical condition that the total pressure loss equals the bubble point pressure. To consider the injection velocity non-uniformity in the *z*-direction, i.e., 2D flow model^26^, the velocity in Eq. (6) should be optimized by the line-averaged injection velocity using the aforementioned non-uniformity coefficient *δ*. The average injection velocity in a 2D model can be written as

7 $$v_{{{{\mathrm{avg}}}} - {{{\mathrm{center}}}}} = \frac{{v_{{{{\mathrm{max}}}}}}}{{\delta \cdot H_{{{{\mathrm{wetted}}}}} + 1}}.$$

Then the critical flow rate by a 2D model is calculated as follows

8 $$Q_{{{{\mathrm{cr}}}} - 2{{{\mathrm{D}}}}} = v_{{{{\mathrm{avg}}}} - {{{\mathrm{center}}}}} \cdot A_{{{\mathrm{c}}}}.$$

Moreover, the non-uniformity coefficient *λ* in the *x*-direction can be utilized to optimize the average injection velocity further by considering the 3D flow. According to Eqs. (3) and (7), the face-averaged injection velocity in the 3D model can be expressed as

9 $$v_{{{{\mathrm{avg}}}}} = \frac{{v_{{{{\mathrm{avg}}}} - {{{\mathrm{center}}}}}}}{{\lambda + 1}} = \frac{{v_{{{{\mathrm{max}}}}}}}{{\left( {\lambda + 1} \right) \cdot \left( {\delta \cdot H_{{{{\mathrm{wetted}}}}} + 1} \right)}}.$$

Then the critical flow rate as predicted by a 3D model is calculated as follows

10 $$Q_{{{{\mathrm{cr}}}} - 3{{{\mathrm{D}}}}} = v_{{{{\mathrm{avg}}}}} \cdot A_{{{\mathrm{c}}}}.$$

**Fig. 8:**
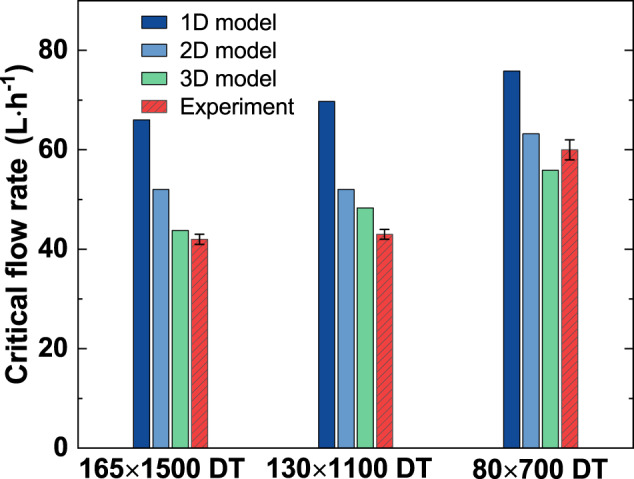
The critical flow rates in different prediction models and experiments. (The error bar indicates the standard deviation of four independent experiments).

Figure 8 compares the critical flow rates predicted by these models and that measured by experiments. The non-uniformity coefficients are determined from the injection velocity fields as discussed above, and their values corresponding to the experimental condition are listed in Supplementary Table 5. In the experiments, the critical flow rate is determined as the flow rate when the first bubble flows across the mesh. With regard to the LAD channel design, the 3D model, which considers the injection velocity non-uniformity in both *z* and *x* directions, performs the best in predicting the critical flow rate. The average deviations between the 3D, 2D, and 1D models and the experiments are 8.4%, 16.7%, and 48.5%, respectively. It should be noted that the non-uniformity coefficients may be influenced by the geometry of the channel and the working fluids, which should be further analyzed in the future. Nevertheless, the present results prove that the velocity distributions in both streamwise and spanwise directions of the channel flow affect the critical flow rate of the screen channel LAD, and neglecting the 3D injection effect would overpredict the critical flow rate.

### The influence of gravity

**Fig. 9:**
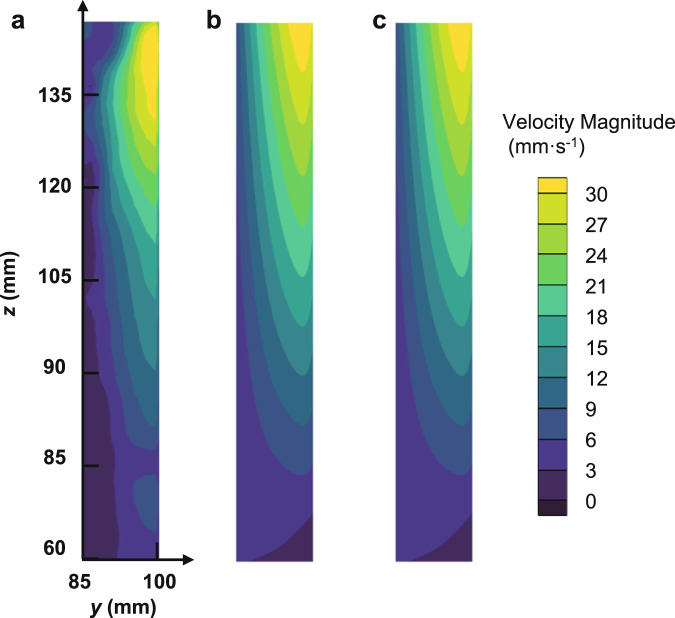
The velocity distribution in the LAD channel for 80 × 700 DT at *Q* = 43 L h^−1^. **a** Experiment, **b** simulation at *g*_*z*_ = −9.8 m s^−2^, and **c** simulation at *g*_*z*_ = 0 m s^−2^.

In order to quantify the influence of gravity, simulation cases for single-phase outflow in the LAD channel were tested at *g*_*z*_ = 0 m s^−2^. Figure 9 shows the velocity distributions in the LAD channel from the experiment and from simulations at *g*_*z*_ = −9.8 m s^−2^ and *g*_*z*_ = 0 m s^−2^. For single-phase flow, gravity has little influence on the velocity distribution inside the channel. However, the critical flow rate is reduced under normal gravity due to hydrostatic pressure loss (Supplementary Equation 4).

Moreover, microgravity changes the distribution of the vapor and liquid phases outside the channel. Liquid tends to gather near the tank walls and vapor tends to concentrate in the middle of the tank^2^. Therefore, the middle of the LAD channel is more likely to be exposed to vapor, which introduces a slight change in the velocity distribution in the channel. For a wetted porous woven mesh in continuous contact with bulk liquids, the non-uniformity coefficient can also be utilized directly to evaluate the average injection velocity. In the region exposed to vapor, there is no mass transported and the pressure decreases mainly due to friction loss. Therefore, the injection velocity non-uniformity could be enlarged by the random vapor-liquid interface. Nevertheless, the injection velocity non-uniformity still plays a major role in the overall pressure distribution inside the channel, and on-ground experimental investigation provides essential guidance for the design of screen channel LADs for on-orbit missions^29^.

## Discussion

In summary, we investigated the flow characteristics at the entrance region of a screen channel liquid acquisition device (LAD) in this study. An anti-gravity liquid acquisition system comprising a test tank, PIV facility, and data acquisition system was built. The influences of various meshes (80 × 700 DT, 130 × 1100 DT, 165 × 1500 DT) and flow rates (0–60 L h^−1^) were investigated in detail. Numerical simulations of single-phase outflow in the LAD channel were also conducted at *g*_*z*_ = 0 m s^−2^ and *g*_*z*_ = −9.8 m s^−2^ using a porous-jump model for the porous woven mesh. The injection velocity non-uniformity in the *x* and *z* directions were studied in detail, and a prediction model was proposed to evaluate the critical flow rate of the screen channel LAD based on the three-dimensional injection flow fields. The main conclusions are as follows:

1. For single-phase liquid flow, the injection velocity is almost perpendicular to the porous woven mesh. The velocity increases along the channel flow direction and reaches its maximum near the outlet. In the spanwise direction, the velocity distribution is of approximately parabolic profile.
2. Experimental results indicate that porous woven meshes with smaller pores produce less uniform injection velocities along the channel flow direction. The maximum injection velocity non-uniformity may reach 30% for a 165 × 1500 DT mesh. In the spanwise direction, the velocity is not sensitive to the variation of the mesh specification. For the flow rate range considered in this study, the variation of flow rate has a negligible influence on velocity non-uniformity in both directions.
3. A 3D model was proposed to predict the critical flow rate of the screen channel LAD, which considers the injection velocity non-uniformity in both streamwise and spanwise directions. The results of the 3D model were compared to experimental data to reveal an error of less than 8.4%, which indicates that the model is reasonable. In particular, the accuracy of the 3D model is much better than that of the 1D and 2D models.
4. Microgravity influences the location of the vapor-liquid interface due to the dominance of the capillary force. Thus, the middle of the LAD channel may be more likely to be exposed to vapor in microgravity conditions. However, simulation results indicate that gravity has little influence on the velocity distribution for the wetted region and inside the channel for single-phase flow.

Based on the present study, we also suggest reducing the injection velocity non-uniformity by improving the LAD design. Possible approaches include optimizing the position of the LAD channel outlet, optimizing the geometry of the LAD channel, and using combinations of porous woven mesh types. Moreover, outflow tests using various fluids, with various channel sizes, for a larger range of flow rates, and under microgravity conditions, should be conducted in future work to validate and improve the present model further.

## Methods

### Theoretical basis

The primary parameters that govern LAD performance are the bubble point pressure and the total pressure loss. The bubble point pressure^29,30^ can be expressed using the Young–Laplace equation,

11 $${{\Delta }}P_{{{{\mathrm{BP}}}}} = 4\gamma {{{\mathrm{cos}}}}\theta _{{{\mathrm{c}}}}/D_{{{\mathrm{p}}}},$$

where *γ* is the surface tension of the fluid; $\theta _{{{\mathrm{c}}}}$ is the contact angle; and $D_{{{\mathrm{p}}}}$ is the effective pore diameter of the porous woven mesh.

The total pressure loss inside the LAD channel should be less than ${{\Delta }}P_{{{{\mathrm{BP}}}}}$ to prevent vapor penetration into the channel. For the experiments of Fig. 1a, The total pressure loss (${{\Delta }}P_{{{{\mathrm{total}}}}}$)^10^ is expressed as

12 $${{\Delta }}P_{{{{\mathrm{total}}}}} = {{\Delta }}P_{{{{\mathrm{hydrostatic}}}}} + {{\Delta }}P_{{{{\mathrm{FTS}}}}} + {{\Delta }}P_{{{{\mathrm{friction}}}}} + {{\Delta }}P_{{{{\mathrm{dynamic}}}}} + {{\Delta }}P_{{{{\mathrm{other}}}}},$$

where ${{\Delta }}P_{{{{\mathrm{hydrostatic}}}}}$ is the hydrostatic pressure in the LAD channel, ${{\Delta }}P_{{{{\mathrm{FTS}}}}}$ is the FTS pressure drop, ${{\Delta }}P_{{{{\mathrm{friction}}}}}$ is the frictional loss inside the LAD channel, ${{\Delta }}P_{{{{\mathrm{dynamic}}}}}$ is the dynamic pressure drop, and ${{\Delta }}P_{{{{\mathrm{other}}}}}$ is the pressure loss caused by vibration and fluid sloshing. In microgravity environments, ${{\Delta }}P_{{{{\mathrm{FTS}}}}}$ dominates the pressure loss term and influences the operational efficiency directly since the hydrostatic pressure is negligible^26^.

According to Wang et al.^24^, the FTS pressure drop can be expressed as

13 $${{\Delta }}P_{{{{\mathrm{FTS}}}}} = A_{{{\mathrm{p}}}}\mu v + B_{{{\mathrm{p}}}}\rho v^2,$$

where $A_{{{\mathrm{p}}}}$ and $B_{{{\mathrm{p}}}}$ are FTS coefficients, which are determined by the geometry of the porous woven mesh; *μ* is the fluid viscosity; and $\rho$ is the fluid density.

The maximum allowable liquid flow flux is referred to as the critical flow rate ($Q_{{{{\mathrm{cr}}}}}$) and occurs when the total pressure loss is equal to the bubble point pressure (${{\Delta }}P_{{{{\mathrm{total}}}}} = {{\Delta }}P_{{{{\mathrm{BP}}}}}$). Since the traditional 1D model assumes that the velocity across the porous woven mesh is uniform^10,23,25,31^, the critical flow rate of the LAD could be calculated usind Eq. (6). However, recent studies have indicated a large disparity between the critical flow rate derived from the 1D and experimental data, since the distribution of the fluid injection velocity at the porous mesh plays an important role in the critical flow rate, but was less considered in the previous studies^26^.

### Experimental setup

All the outflow tests of screen channel LAD were conducted in a transparent test tank made from quartz glass, a material that exhibits good chemical stability and excellent optical properties (Supplementary Fig. 1). The size of the tank was 200 × 200 × 210 mm^3^. The upper side of the test tank was a stainless-steel plate affixed to the LAD channel with epoxy resin. It could also be removed from the test tank so that the LAD channel could be replaced and tests run under different conditions. The test system was placed at ground level with *g*_*z*_ = −9.8 m s^−2^.

The LAD channel was a hollow duct composed of three transparent walls and a porous wall made from porous metal mesh. The LAD channel was 15 mm long (*L*), 15 mm wide (*W*), and 200 mm high (*H*). The porous wall of the LAD channel was 85 mm away from the inner wall of the tank. Preliminary experiments indicated that the distance had a negligible effect on the critical flow rate provided that the distance was larger than 10 mm, as the pressure loss caused by the distance between the porous wall and the inner wall is much smaller than the other items in Eq. (12). The bottom of the LAD channel is about 5 mm away from the wall of the tank. Considering the injection velocity is parallel to the bottom wall, and the velocity magnitude is very low at the dead-end region, the distance is assumed to have no significant effect on the LAD performance^21^. Assembly of the LAD channel proceeded as follows. Prior to the test, the porous woven mesh was cleaned in an ultrasonic cleaner using alcohol and deionized water. The porous woven mesh was affixed to the channel using epoxy resin to avoid sealing and delamination at the edges. Two differential pressure transducers were mounted 25 mm apart at the bottom and top of the channel, as shown in Fig. 1c.

Dutch Twill screens were used in the experiments since this type of mesh offers the smallest pore diameter and the most tortuous flow path. These features can prevent vapor ingestion^10^. Three porous woven meshes with different pore sizes were chosen as experimental materials: an 80 × 700 Dutch Twill (DT) mesh, a 130 × 1100 DT mesh, and a 165 × 1500 DT mesh. The porous meshes are labeled in the form $n_{{{\mathrm{w}}}} \times n_{{{\mathrm{s}}}}$, where $n_{{{\mathrm{w}}}}$ and $n_{{{\mathrm{s}}}}$ denote the number of warp and shute wires per inch, respectively.

Prior to the experiments, the bubble point pressures of the meshes were tested^32^, and the effective pore diameter *D*_p_ was obtained based on Eq. (11) (Supplementary Methods, Supplementary Tables 2–4). The effective pore diameters overlap with historical values^33^ considering the uncertainties caused by measurement and manufacturing. The diameters of wrap and shute wires were obtained from scanning electron microscopy (SEM, by TESCAN VEGA3) images as shown in Supplementary Fig. 2, while the porosity *ε*, specific surface area *S*_v_, hydraulic pore diameter *D*_h_ (=4*ε*/*S*_v_), and the FTS pressure coefficients in Eq. (13) were calculated according to the method of Wang et al.^24^ from the pore geometries. The relevant parameters and the geometric properties of the test meshes are listed in Table 1.

The PIV system (LaVision Inc.) included a high-speed camera (4008 × 2672 pixels) and a laser pulse generator (Nd:YAG LASER NANO TRL, 425 mJ at 532 nm and 10 Hz). Particle motion was captured by operating the high-speed camera at 2 Hz. The test tank and the areas recorded using the PIV technique were shown in Fig. 1c. The recorded area at *yz* plane starts at *x* = 105 mm, *y* = 5 mm, and *z* = 60 mm. With a 90% fill level, the vapor-liquid interface and channel bottom were located at *z* = 180 mm and *z* = 0 mm, respectively. The laser was mounted perpendicular to the porous woven mesh at *x* = 105 mm, and thus the camera captured particle motion across the *yz* plane of the porous mesh. Due to the limited field-of-view, the size of the area recorded by the camera was limited to 80 × 165 mm. To test the spanwise velocity distribution, three *xy* planes at *z* = 40 mm, 100 mm and 160 mm were recorded. The orientations and positions of the high-speed camera and the laser pulse generator were adjusted accordingly. Deionized water was chosen as the test fluid (*ρ* = 998.2 kg m^−3^, *μ* = 1.002 mPa s, and *γ* = 72.8 mN m^−1^). Prior to the experiments, water was doped with particles with diameters of 9 μm and density of 1.1 g cm^−3^, so that the particles’ volume fraction is around 0.002%. At such low concentrations, the tracking particles have been verified to have no obvious effect on the water properties, including density, surface tension and viscosity (<5%)^34,35^. Experiments were conducted to recheck the difference in bubble point pressures and contact angles between deionized water and doped water. The difference in bubble point pressures was found to be within the experimental uncertainty. The contact angle of deionized water and doped water show the same value of 71 (±2)° on the stainless-steel plate (Supplementary Fig. 3). Preliminary analysis also indicated that the particles would not obviously clog the porous woven mesh during the tests^36^ (Supplementary Fig. 4) . A peristaltic pump was used to drain water from the channel and pumping it back into the test tank to maintain the fill level. The flow rate of the peristaltic pump could also be adjusted to achieve different working conditions.

### Data acquisition and reduction

Experiments were conducted at different flow rates for each type of woven porous mesh. All of the test cases were repeated four times to verify the consistency and repeatability, with each test conducted at the steady state. The images captured using the camera were analyzed via a cross-correlation algorithm using the LaVision PIV module and post-processed in MATLAB. The pressure drop was detected using a pressure difference sensor with a range of 0–20 kPa. The flow rate was detected using two flow meters with ranges of 4–40 L h^−1^ and 40–400 L h^−1^, respectively. The uncertainty of the pressure difference sensor is 0.075%, while the uncertainty of the flow rate is 1.5%. The diameter of particles is 1.7–1.9 pixels in the tracking images, and the measurement uncertainty is between 0.05 and 0.1 px^37^. Therefore, the uncertainty of the PIV-derived velocity vectors is below 5.9%.

### Reporting summary

Further information on research design is available in the Nature Research Reporting Summary linked to this article.

## Supplementary information

Supplementary Information

Reporting Summary

## Data Availability

All data generated or analyzed during this study are included in this published article and its supplementary information files.

## Code availability

The code used to generate the plots are available from the corresponding author on reasonable request.
