# Supplementary material for: A three-dimensional flow model of screen channel liquid acquisition devices for propellant management in microgravity
Source: NPJ Microgravity. 2022 Jul 28;8:28. doi: 10.1038/s41526-022-00216-5 (PMC9334641; doi:10.1038/s41526-022-00216-5)
Supplement: Supplementary file 1 — Supplementary Information [file 41526_2022_216_MOESM1_ESM.docx]

**Supplementary Information**

A three-dimensional flow model of screen channel liquid acquisition devices for propellant management in microgravity

Zheng Wang,^1^ Guang Yang,^1, 3,^ Ye Wang,^1^ Xin Jin,^2,3^ Rui Zhuan,^2,3^ Hao Zhang,^2,3^ Jingyi Wu^1,3^

^1^*Institute of Refrigeration and Cryogenics, Shanghai Jiao Tong University, Shanghai 200240, China*

^2^*Aerospace System Engineering Shanghai, Shanghai 201109, China*

^3^*Joint Laboratory for Cryogenic Propulsion Technology of Aerospace Systems, Shanghai 200240, China*

**Contents**

[Supplementary Methods 3](#_Toc106642458)

[1. Calculation of the effective pore diameter from the bubble point pressures 3](#_Toc106642459)

[2. Calculation of velocity distribution using 2D Navier-Stokes equations 4](#_Toc106642460)

[Supplementary Figures 5](#_Toc106642461)

[Supplementary Figure 1. A close view of the LAD channel and tank. 5](#_Toc106642462)

[Supplementary Figure 2. Scanning electron microscopy images of the meshes. 6](#_Toc106642463)

[Supplementary Figure 3. Contact angle of water on a stainless-steel plate 7](#_Toc106642464)

[Supplementary Figure 4. Particle densities inside and outside the screen channel 8](#_Toc106642465)

[Supplementary Figure 5. A vertically oriented rectangular LAD channel for the theoretical analysis of velocity distribution in the *z* direction. 9](#_Toc106642466)

[Supplementary Tables 10](#_Toc106642467)

[Supplementary Table 1. Nomenclature. 10](#_Toc106642468)

[Supplementary Table 2. Calculation of the effective pore diameter of 80$\times$700 DT 11](#_Toc106642469)

[Supplementary Table 3. Calculation of the effective pore diameter of 130$\times$1100 DT 11](#_Toc106642470)

[Supplementary Table 4. Calculation of the effective pore diameter of 165$\times$1500 DT 11](#_Toc106642471)

[Supplementary Table 5. Non-uniformity coefficients for various porous woven meshes. 12](#_Toc106642472)

[Supplementary Discussion 13](#_Toc106642473)

[Theoretical analysis of the velocity distribution in the *z* direction 13](#_Toc106642474)

[Supplementary References. 15](#_Toc106642475)

# Supplementary Methods

## 1. Calculation of the effective pore diameter from the bubble point pressures

The effective pore diameter of the porous woven mesh was calculated with *D*_p_=4*γ*cos*θ*_c_/Δ*P*_BP_ using the experimentally measured bubble point pressures. Deionized water ($\rho$=998.2 kg·m^-3^, $\mu$=1.002 mPa·s, and $\gamma$=72.8 mN·m^-1^) was used as the test liquid. The contact angle was measured using the sessile droplet method by the contact angle measuring instrument (Instrumental Analysis Center of Shanghai Jiao Tong University, DSA100). The value of the contact angle of water on a stainless-steel plate was 71 (±2)°, as determined from four independent trials, considering the fitting errors (Supplementary Figure 3).

The bubble point pressures of various screens were tested using a home-made device ^[1]^. At least four independent tests were conducted for each mesh. The uncertainty of the effective pore diameter ($U_{D_{p}}$) was determined using the standard procedures as follows:

$U_{D_{p}}^{2}=U_{\cos\theta}^{2}\left( \frac{4\gamma\sin\theta}{\Delta P_{\mathrm{BP}}} \right)^{2}+U_{\Delta P_{\mathrm{BP}}}^{2}\left( \frac{4\gamma\cos\theta}{{\Delta P_{\mathrm{BP}}}^{2}} \right)^{2}$ (1)

Results of the bubble point pressures, the effective pore diameters, and their uncertainties are shown in Supplementary Tables 2-4, for the mesh types of 80$\times$700 DT, 130$\times$1100 DT and 165$\times$1500 DT, respectively.

## 2. Calculation of velocity distribution using 2D Navier-Stokes equations

The calculation procedure of the injection velocity distribution in the *z*-direction using the 2D Navier-Stokes equations, as derived by Darr et al. ^[2]^, is introduced in this section, for the present experimental condition at $Q$=43 L·h^-1^ with 80$\times$700 DT mesh.

In the model of Darr et al. ^[2]^, the variation of velocity inside the LAD channel towards the outflow direction was described by the variable *f*, as follows (Eq. 16 in Darr et al. ^[2]^):

$v_{\mathrm{injection}}=-\bar{u_{e}}\frac{df}{dz^{*}}$ (2)

where $\bar{u_{e}}=\dot{\frac{m_{e}}{\rho WL}}$ was the mean velocity along outflow direction at the LAD channel exit, and the *z*^*^ (=*z*/*L*) was the height dimensions normalized by *L*.

The variable *f* can be obtained by solving the following second-order nonlinear ordinary differential equation (Eq. 28 in Darr et al. ^[2]^):

$\left( \frac{1}{Re_{e}}+\frac{N_{\mathrm{lam}}}{Re_{e}}+2C_{\mathrm{turb}}f^{'} \right)f^{''}-\frac{91.46}{Re_{e}}\left( 1+f \right)-2.348f^{'}\left( 1+f \right)=0$ (3)

where $Re{}_{e}=\rho\bar{u_{e}}L/\mu$, $N_{\mathrm{lam}}=C_{\mathrm{lam}}L$, $C_{\mathrm{turb}}=\frac{\beta\varphi B}{\varepsilon^{2}D_{p}}$, $C_{\mathrm{lam}}=\frac{S_{v}\varphi{Ba}^{2}}{\varepsilon^{2}}$. The boundary conditions were *f* (0) = -1 and *f* (*H*^*^) = 0. According to Darr et al. ^[2]^, values of 4.2 and 0.2 were used for $\alpha$ and $\beta$, respectively. The parameters $\varphi$*,* $\varepsilon$, *D*_p_, *B* and $S_{v}$ were the tortuosity factor, porosity, effective pore diameter, screen thickness and surface area to unit volume ratio, respectively. The parameters for the 80$\times$700 DT mesh could be found in Table 1 or determined by the method of Wang et al. ^[3]^ from the 3D pore geometries (Supplementary Figure 2). *ρ*, *μ*, *L*, *W* and *H* were the density of fluid, viscosity of the fluid, length, width and height of the LAD channel.

Using the input parameters mentioned above, the Supplementary equation (3) was numerically solved by MATLAB. The velocity distribution along the *z*-direction, as plotted in Figure 7a, was obtained.

# Supplementary Figures

**1) The LAD channel and fluid tank in the experimental setup**


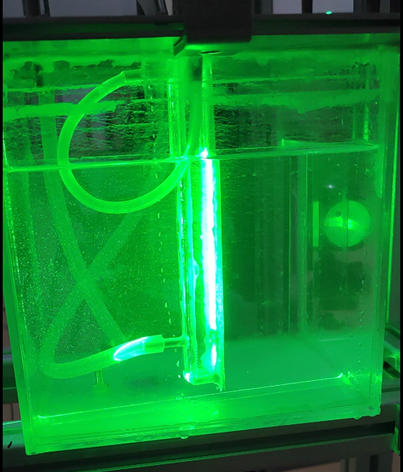


## Supplementary Figure 1. A close view of the LAD channel and fluid tank.

The size of the tank was 200×200×210 mm^3^. The size of the LAD channel was 15 ×15 × 200 mm^3^.

**2) Scanning electron microscopy (SEM) images of of the meshes**

The SEM images of the woven meshes were obtained by TESCAN VEGA3, at the Instrumental Analysis Center of Shanghai Jiao Tong University.


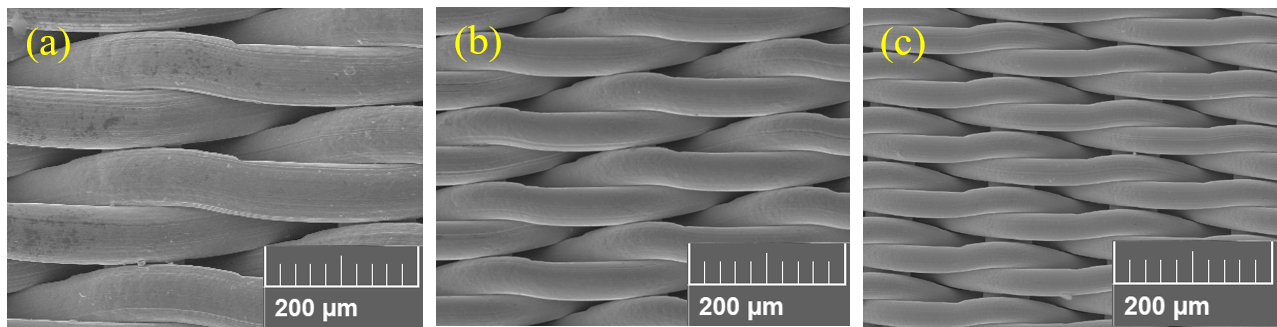


## Supplementary Figure 2. Scanning electron microscopy images of the meshes.

(a) 80$\times$700 DT, (b) 130$\times$1100 DT, and (c) 165$\times$1500 DT.

**3) Measurement of contact angle**


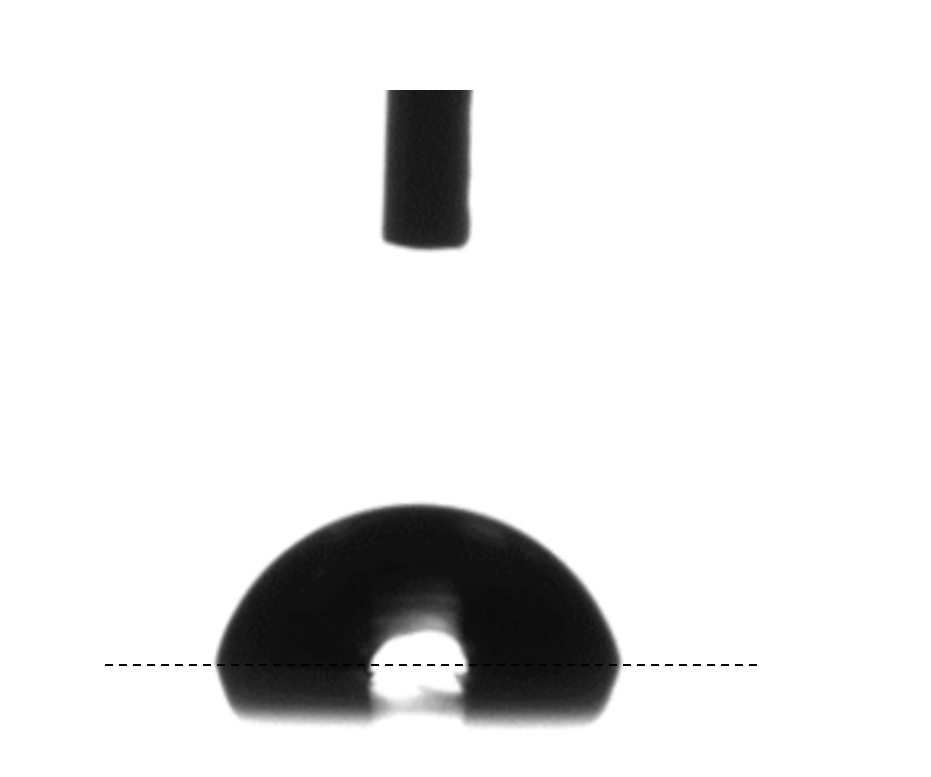


## Supplementary Figure 3. Contact angle of water on a stainless-steel plate

**4) Discussion on the clogging effect of the tracking particles**

As the clogging effect of the tracking particles might be an issue for the PIV experiments with a woven mesh involved, a preliminary test was conducted to check if the doping could influence the measurements. Firstly, we compared the concentration of particles inside and outside the screen channel from the captured images (Supplementary Figure 4). With the same area of 5 mm×5 mm, the particle numbers inside and outside the LAD channel were 16 (±2) and 15 (±3), respectively. So we can assume that the particles can almost flow across the mesh screen freely during the experiments. Secondly, the ratio of pore diameter to particle diameter was around 3~ 5 in our experiments. According to Marin et al.^[4]^, the fluid flow would not be clogged by particles when the ratio of pore diameter to particle diameter is larger than 3. Therefore, we can consider that the doping particles would not significantly affect the fluid flow through the porous woven mesh during the experiments.


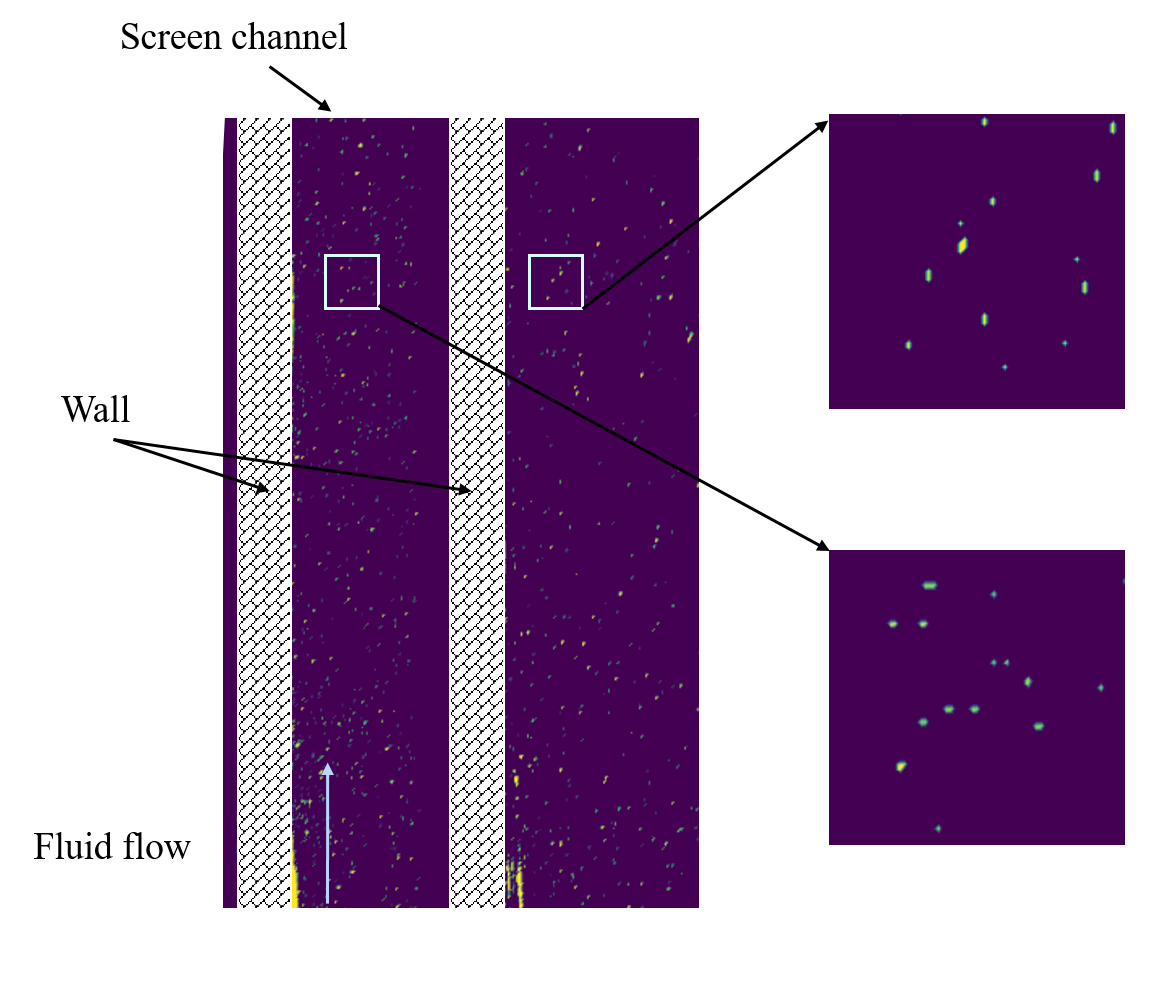


## Supplementary Figure 4. Particle densities inside and outside the screen channel

(The mesh is in the position between the two walls and facing back to the reader.)

**5) Schematic of a vertically oriented rectangular LAD channel for the theoretical analysis of velocity distribution**


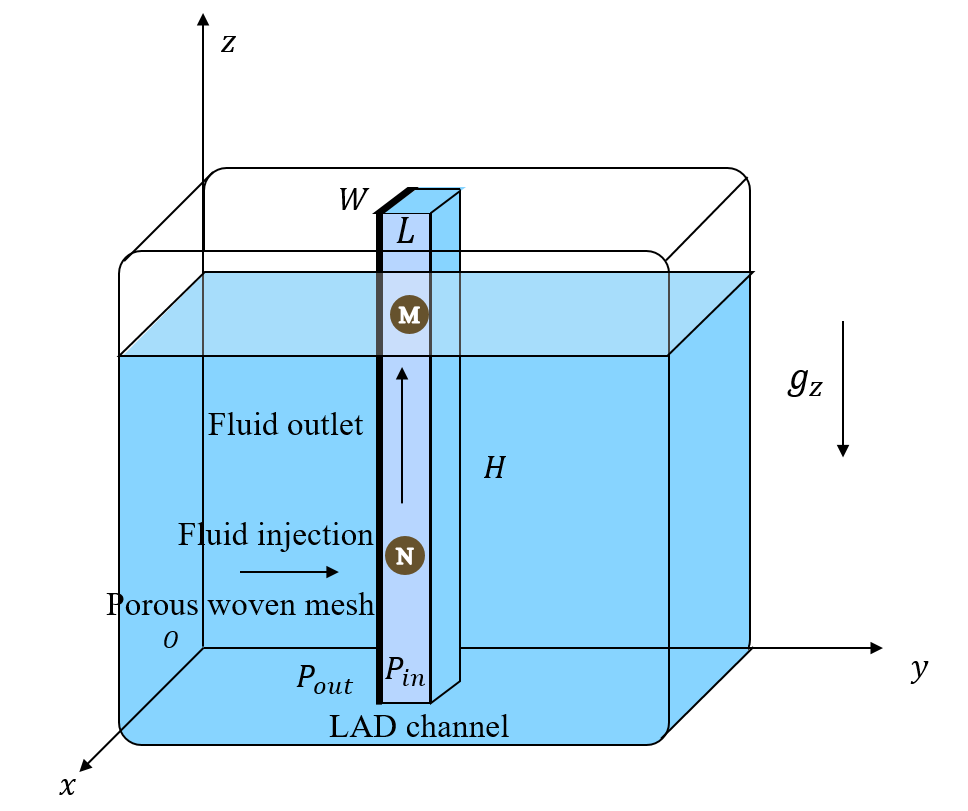


## Supplementary Figure 5. A vertically oriented rectangular LAD channel for the theoretical analysis of velocity distribution in the *z* direction.

# Supplementary Tables

## Supplementary Table 1. Nomenclature.

| $A_{c}$ | effective flow area of the porous woven mesh (m^2^) | $\gamma$ | surface tension (mN·m^-1^) |
| --- | --- | --- | --- |
| $A_{f},$ $B_{f}$ | frication resistance coefficient | $\theta_{c}$ | contact angle (°) |
| $A_{d}$ | dynamic pressure drop coefficient | $\rho$ | density of the liquid (kg·m^-3^) |
| $A_{p}$ | first-order-term FTS coefficient (m^-1^) | $\delta$ | non-uniformity coefficient in the *z* direction (m^-1^) |
| $B_{p}$ | second-order-term FTS coefficient | $\lambda$ | non-uniformity coefficient in the *y* direction |
| $D_{y}$ | viscous resistance coefficient | $\mu$ | liquid viscosity (mPa$\cdot$s) |
| $C_{y}$ | inertial resistance coefficient | $v$ | velocity in the *y* direction (m·s^-1^) |
| $D_{h}$ | hydraulic pore diameter (μm) | $u$ | velocity in the *z* direction (m·s^-1^) |
| $D{}_{p}$ | effective pore diameter (μm) | $g$ | gravity acceleration (m·s^-2^) |
| $H$ | height of the LAD channel (mm) | $x, y, z$ | Cartesian coordinates |
| $L$ | length of the LAD channel (mm) | $\Delta P_{\mathrm{FTS}}$ | flow-through-screen pressure drop (kPa) |
| $W$ | width of the LAD channel (mm) | $\Delta P_{\mathrm{frictional}}$ | frictional pressure loss inside LAD channel (kPa) |
| $Q$ | volumetric flow rate (L·h^-1^) | $\Delta P_{\mathrm{dynamic}}$ | dynamic pressure loss inside LAD channel (kPa) |
| $n_{w}$ | number of warp wires per inch | $\Delta P_{\mathrm{BP}}$ | bubble point pressure (kPa) |
| $n_{s}$ | number of shute wires per inch | $S_{v}$ | specific surface area (m^-1^) |

## Supplementary Table 2. Calculation of the effective pore diameter of 80$\boldsymbol{\times}$700 DT

| **test no.** | **Δ*P*_BP_ (kPa)** | **average Δ*P*_BP_ (kPa)** | $\boldsymbol{U}_{\boldsymbol{\Delta}_{\mathbf{BP}}}$ **(kPa)** | ***D*_p_ (μm)** | $\boldsymbol{U}_{\boldsymbol{D}_{\mathbf{p}}}$ **(μm)** |
| --- | --- | --- | --- | --- | --- |
| 1 | 1.682 | 1.757 | 0.0713 | 53.4 | 5.8 |
| 2 | 1.693 |  |  |  |  |
| 3 | 1.809 |  |  |  |  |
| 4 | 1.846 |  |  |  |  |

## Supplementary Table 3. Calculation of the effective pore diameter of 130$\boldsymbol{\times}$1100 DT

| **test no.** | **Δ*P*_BP_ (kPa)** | **average Δ*P*_BP_ (kPa)** | $\boldsymbol{U}_{\boldsymbol{\Delta}_{\mathbf{BP}}}$ **(kPa)** | ***D*_p_ (μm)** | $\boldsymbol{U}_{\boldsymbol{D}_{\mathbf{p}}}$ **(μm)** |
| --- | --- | --- | --- | --- | --- |
| 1 | 2.520 | 2.667 | 0.0911 | 35.2 | 3.7 |
| 2 | 2.732 |  |  |  |  |
| 3 | 2.753 |  |  |  |  |
| 4 | 2.662 |  |  |  |  |

## Supplementary Table 4. Calculation of the effective pore diameter of 165$\boldsymbol{\times}$1500 DT

| **test no.** | **Δ*P*_BP_ (kPa)** | **average Δ*P*_BP_ (kPa)** | $\boldsymbol{U}_{\boldsymbol{\Delta}_{\mathbf{BP}}}$ **(kPa)** | ***D*_p_ (μm)** | $\boldsymbol{U}_{\boldsymbol{D}_{\mathbf{p}}}$ **(μm)** |
| --- | --- | --- | --- | --- | --- |
| 1 | 3.268 | 3.132 | 0.0872 | 29.9 | 3.1 |
| 2 | 3.112 |  |  |  |  |
| 3 | 3.025 |  |  |  |  |
| 4 | 3.123 |  |  |  |  |

## Supplementary Table 5. Non-uniformity coefficients for various porous woven meshes (fill level: 90%, channel size: 15 mm$\boldsymbol{\times}$15 mm$\boldsymbol{\times}$200 mm).

| Mesh type | 80$\times$700 DT | 130$\times$1100 DT | 165$\times$1500 DT |
| --- | --- | --- | --- |
| $\delta{⸱H}_{\mathrm{wetted}}$ | 17.1% | 22.9% | 29.2% |
| $\lambda$ | 16.0% | 16.3% | 16.5% |

# Supplementary Discussion

## Theoretical analysis of the velocity distribution in the *z* direction

For the vertically oriented rectangular LAD channel with length *L*, width *W*, and height *H* shown in Supplementary Figure 5, the distribution of injection velocity in the *z-*direction is governed by the variation of pressure in the channel. Considering the experimental condition of upward flow, the pressure difference between any two locations M and N in the channel can be written as

$\Delta{P_{\mathrm{in}}}^{\text{M - N}}=P_{\text{friction}}\left( M \right)-P_{\text{friction}}\left( N \right)+P_{\text{dynamic}}\left( M \right)-P_{\text{dynamic}}\left( N \right)+\rho g_{z}\Delta h^{\text{M - N}}$ (4)

where $\Delta P_{\mathrm{in}}$ is the pressure difference inside the channel, $g_{z}$ is the gravitational acceleration and $\Delta h$ is the height difference between M and N*.*

The pressures inside and outside the LAD channel at the bottom (*z*=0) are $P_{0}$ and $P_{1}$, respectively. Thus, the local pressure inside the LAD channel at position *z* can be expressed as

${P^{z}}_{\mathrm{in}}=P_{0}-P_{\text{friction}}\left( z \right)-P_{\text{dynamic}}\left( z \right)-\rho g_{z}\Delta h^{z}$ (5)

Correspondingly, the local pressure outside the LAD channel at position *z* is expressed as

${P^{z}}_{\mathrm{out}}=P_{1}-\rho g\Delta h^{z}$ (6)

Then, the FTS pressure drop at any position can be calculated as follows:

$\Delta P_{\text{FTS}}={P^{z}}_{\mathrm{out}}-{P^{z}}_{\mathrm{in}}\text{=}\text{Δ}P_{\text{FTS-0}}+P_{\text{friction}}(z)+P_{\text{dynamic}}(z)$ (7)

$\Delta P_{\text{FTS-0}}=P_{1}-P_{0}$ (8)

where $\Delta P_{\mathrm{FTS}-0}$ is the FTS drop at the bottom of the LAD channel. It should be noted that $\Delta P_{\mathrm{FTS}}$ is independent of gravitational acceleration, as the gravity term is eliminated during the subtraction in Supplementary Eq. (7). It is obvious that the FTS pressure drop increases along the flow direction. The increasing pressure difference across the porous woven mesh produces a higher injection velocity near the outlet.

Moreover, the FTS pressure drop is related to the injection velocity and can be calculated using Eq. (13) in the paper (Δ*P*_FTS_ = *A*_p_*μv*+*B*_p_*ρv*^2^). According to Hartwig *et al.*^[5]^, the friction resistance and dynamic pressure drop can be calculated as follows:

$\Delta P_{\text{friction}}\left( z \right)=A_{f}u\mu z+B_{f}u^{2}\rho z$ (9)

$\Delta P_{\text{dynamic}}=\frac{\rho u^{2}}{A_{d}}$ (10)

where $A_{f}$ and $B_{f}$ are friction resistance coefficients, which are controlled by the shape, size, and roughness of the LAD channel, *u* is the velcocity along outflow direction inner LAD channel and $A_{d}$ is the dynamic pressure drop coefficient, which is also controlled by the size of the LAD channel. Consequently, Supplementary Eq. (7) can be rewritten as

$A_{p}\mu\Delta v+B_{p}\rho\Delta v^{2}=A_{f}\mu z\Delta u+B_{f}\rho z\Delta u^{2}+\frac{\rho\Delta u^{2}}{A_{d}}$ (11)

Continuity of mass requires that the fluid flowing through the porous woven mesh be equal to that which flows out of the channel:

$W\int_{x=W/2} v\cdot dz=W\cdot L\cdot u$ (12)

Plugging Supplementary Eq. (12) into Supplementary Eq. (11) allows the pressure balance equation to be expressed as

$A_{p}\mu dv+2B_{p}\rho vdv=\frac{A_{f}\mu z}{L}vdz+\frac{2B_{f}\rho z}{L}uvdz+\frac{2\rho uvdz}{A_{d}L}$ (13)

After simplifying Supplementary Eq. (13), the relationship between the injection velocity and the flow length can be determined by

$(A_{p}\cdot\frac{\mu}{\rho}+2B_{p}v)\cdot\frac{dv}{dz}=\frac{A_{f}vz}{L}\cdot\frac{\mu}{\rho}+\frac{2B_{f}uvz}{L}+\frac{2uv}{A_{d}L}$ (14)

where $dv/dz$ is the non-uniformity coefficient $\delta$, which represents the velocity non-uniformity in *z*-direction. The parameters in Supplementary Eq. (14), including the fluid properties, mesh type, and LAD channel size, shape, and roughness affect the non-uniformity coefficient.

When the woven mesh becomes finer, the terms to the right of the equal sign in Supplementary Eq. (14) would increase, while the term in the brackets on the left changes very slightly. So among the three types of porous woven mesh, the 165×1500 DT has the highest velocity non-uniformity while the 80×700 DT has the lowest velocity non-uniformity in the streamwise direction.

# Supplementary References.

[1] Wang, Y., Wang, Z., Cheng, X., Yang, G. & Wu, J. Pressure-driven phase separation based on modified porous mesh for liquid management in microgravity. *Langmuir* **38**, 2919-2927 (2022)

[2] Darr, S. R., Camarotti, C. F., Hartwig, J. W. & Chung, J. N. Hydrodynamic model of screen channel liquid acquisition devices for in-space cryogenic propellant management. *Phys. Fluids* **29**, 017101 (2017).

[3] Marin, A., Lhuissier, H., Rossi, M. & Kähler, C. J. Clogging in constricted suspension flows. *Phys. Rev. E* **97**, 021102 (2018).

[4] Wang, Y., Yang, G., Huang, Y., Zhuan, R., Wu, J., Analytical model of flow-through-screen pressure drop for metal wire screens considering the effects of pore structures. *Chem. Eng. Sci.* **229**, 116037 (2021).

[5] Hartwig, J. W., Darr, S. R., McQuillen, J. B., Rame, E. & Chato, D. J. A steady state pressure drop model for screen channel liquid acquisition devices. *Cryogenics* **64**, 260-271 (2014).
